# Bimetallic Co-Based (CoM, M = Mo, Fe, Mn) Coatings for High-Efficiency Water Splitting

**DOI:** 10.3390/ma14010092

**Published:** 2020-12-28

**Authors:** Jadranka Milikić, Aldona Balčiūnaitė, Zita Sukackienė, Dušan Mladenović, Diogo M. F. Santos, Loreta Tamašauskaitė-Tamašiūnaitė, Biljana Šljukić

**Affiliations:** 1 Faculty of Physical Chemistry, University of Belgrade, Studentski trg 12–16, 11158 Belgrade, Serbia; jadranka@ffh.bg.ac.rs (J.M.); dusan.mladenovic@ffh.bg.ac.rs (D.M.); 2 Department of Catalysis, Center for Physical Sciences and Technology, Saulėtekio ave. 3, Vilnius LT-10257, Lithuania; aldona.balciunaite@ftmc.lt (A.B.); zita.sukackiene@ftmc.lt (Z.S.); loreta.tamasauskaite@ftmc.lt (L.T.-T.); 3 Center of Physics and Engineering of Advanced Materials, Instituto Superior Técnico, Universidade de Lisboa, 1049-001 Lisbon, Portugal; diogosantos@tecnico.ulisboa.pt

**Keywords:** electroless deposition, cobalt-based coatings, hydrogen evolution reaction, oxygen evolution reaction, water electrolysis

## Abstract

Bimetallic cobalt (Co)-based coatings were prepared by a facile, fast, and low-cost electroless deposition on a copper substrate (CoFe, CoMn, CoMo) and characterized by scanning electron microscopy with energy dispersive X-ray spectroscopy and X-ray diffraction analysis. Prepared coatings were thoroughly examined for hydrogen evolution reaction (HER) and oxygen evolution reaction (OER) in alkaline solution (1 M potassium hydroxide, KOH) and their activity compared to that of Co and Ni coatings. All five coatings showed activity for both reactions, where CoMo and Co showed the highest activity for HER and OER, respectively. Namely, the highest HER current density was recorded at CoMo coating with low overpotential (61 mV) to reach a current density of 10 mA·cm^−2^. The highest OER current density was recorded at Co coating with a low Tafel slope of 60 mV·dec^−1^. Furthermore, these coatings proved to be stable under HER and OER polarization conditions.

## 1. Introduction

The electrochemical water splitting is a simple method to produce high purity hydrogen (H_2_) through cathodic reaction (hydrogen evolution reaction, HER) and on the other side, oxygen (O_2_) through anodic reaction (oxygen evolution reaction, OER) [1,2,3,4,5]. H_2_ gas produced by water electrolysis could be a great replacement for fossil fuels such as coal and petroleum as its consumption produces no carbon dioxide (CO_2_) or other greenhouse gasses [6]. Hence, the number of studies related to water electrolysis has increased significantly every year during the last decade, aiming to increase the process efficiency and decrease its cost [7,8,9,10,11,12]. OER proceeding via a 4-electron pathway is slower than HER proceeding via a 2-electron pathway [6]. Platinum (Pt) showed the best catalytic activity for HER, while iridium oxide (IrO_2_) and ruthenium oxide (RuO_2_) were presented as the best electrocatalysts for OER in alkaline media with low overpotential [1,13]. The major limitations of these electrocatalysts are their high cost, low abundance, and low catalytic stability. It is very important to find appropriate low-cost electrocatalysts that can improve HER and OER kinetics and efficiency. Transition metal-based electrocatalysts combined with noble metals are presented as good electrocatalysts for both HER and OER in alkaline media [14,15]. Also, transition metal phosphides [16,17], sulfides [18,19], selenides [20,21], carbides [22,23] and nitrides [24] have been tested as bifunctional electrocatalysts for HER and OER.

Strained cobalt(II) oxide (S-CoO) exhibited a Tafel slope of 94 mV·dec^−1^, confirming the Heyrovsky–Volmer HER mechanism [25]. Incorporation of a small amount of copper (Cu) impurities into Co_3_O_4_ precursor led to the formation of Co_3_O_4_–CuO nanowires and resulted in improved conductivity as well as good catalytic activity for HER [26]. This mixed transition metal oxide gave a current density of 10 mA·cm^−2^ at 0.288 V vs. reversible hydrogen electrode (RHE) and Tafel slope of 65 mV·dec^−1^ [26]. Fe- and Mn-doped CoP electrocatalysts were tested for HER in 1 M potassium hydroxide (KOH) with a low overpotential of 163 mV·dec^−1^ observed for HER at CoP–FeP [2]. The excellent activity of CoP–FeP for HER could be a consequence of the optimized electronic structure of Co centers and P upon the introduction of Fe [2]. Co_0.85_Se/nitrogen-doped graphene showed small Tafel slopes of 76.5 mV·dec^−1^ with a low HER onset potential of 111 mV in 1 M KOH [21]. A hybrid electrocatalyst of FeCo alloy nanoparticles deposited on a porous N-doped carbon (FeCo-TA@CMS) was also tested for HER in alkaline media where Tafel slope value of 102 mV·dec^−1^ revealed that the Volmer process is the rate-controlling step [27]. Factors affecting HER activity of bimetallic nickel M_x_Ni_1-x_ (M = Cr, Mo, and W; x = 0.2) alloys were defined by density functional theory (DFT) revealing that OH species on the surface preferred to adsorb on the top site of the M element, which could change the hydrogen adsorption energy (ΔG_H_^*^) on the active site [28].

As for OER, Co-based materials could easily form hydroperoxo (-OOH) species and after that deprotonate to O_2_ formation [29]. Co-, Co_x_Fe-, and Fe-metal-organic frameworks (MOFs) with different Co/Fe molar ratios were tested for OER in 1 M KOH [30]. Co_2_Fe–MOF presented the best electrocatalytic activity for OER with low overpotential at 10 mA·cm^−2^ (280 mV) and low Tafel slope (44.7 mV·dec^−1^) [30]. Ag_2_S–CoS hetero-nanowires showed excellent OER activity with an overpotential of 275 mV, low charge transfer resistance, and high electrical conductivity due to the octahedral Co sites as active sites for the OER in 1 M KOH [6]. Tafel slopes of 52, 66, and 60 mV·dec^−1^ were obtained for OER at the activated multishell Mn–Co oxyphosphide, Mn–Co oxyphosphide, and Mn–Co oxide particles, respectively, in 1 M KOH [16]. These metal phosphide-based materials showed that during the electrochemical process, the oxyphosphides are oxidized to oxide or hydroxide species presenting active sites for OER [16]. Post-phosphorization treatment of NiMoO_4_ (P-NMO) induced new active sites and resulted in improved performance for OER in alkaline solution so that OER onset potential at 5 mA·cm^−2^ (370 mV) was lower than that of IrO_2_ (390 mV) along with lower Tafel slope (70.3 mV·dec^−1^) [31]. A three-dimensional self-operated Co-doped nickel selenide nanoflowers (3D Co–NiSe/NF) electrode showed low OER overpotential with Tafel slope of 111 mV·dec^−1^ in 1 M KOH [32].

Transition metal-based materials, such as molybdenum disulfide (MoS_2_) synthesized by the hydrothermal method, showed to be bifunctional electrocatalysts for water splitting with high HER and OER activities [1]. Furthermore, iron selenide on Ni foam (FeSe–NF) proved to be a good electrocatalyst for HER and OER in 1 M KOH [33] with the lowest OER onset potential (ca. 150 mV) compared to Se–NF (190 mV), Fe–NF (300 mV) and NF (320 mV) [33]. Tafel slopes of 201, 181, 155 and 145 mV·dec^−1^ were obtained during HER at NF, Se–NF, Fe–NF and FeSe–NF, respectively. Ni–Co alloy nanostructured electrodes with four different amounts of Ni and Co were also examined for both HER and OER in KOH aqueous solution. Ni–Co nanowires with ca. 95% of Co and 5% of Ni showed the highest HER and OER activity with a low potential of –0.231 V and 1.494 V required to attain a current density of 10 mA·cm^−2^ for HER and OER, respectively [34].

In this work, Co-based bimetallic coatings (CoM, M = Fe, Mn, Mo), as well as monometallic Co and Ni coatings, were prepared by a facile, fast, and low-cost electroless deposition method. The morphology and composition of prepared coatings were explored by scanning electron microscopy (SEM) with energy dispersive X-ray spectroscopy (EDX), X-ray diffraction analysis and inductively coupled plasma–optical emission spectrometry (ICP–OES) analysis. The effect of combining Co with left-hand side transition metals on activity for HER and OER in alkaline media (1 M KOH) was then systematically studied by linear sweep voltammetry (LSV), cyclic voltammetry (CV), electrochemical impedance spectroscopy (EIS), and chronoamperometry (CA).

## 2. Experimental

### 2.1. Preparation of Coatings

Electroless deposition of Co was performed on the copper (Cu) surface. Prior to electroless deposition of Co, Cu sheets (1 cm × 1 cm) were pre-treated with 50–100% calcium magnesium oxide, known as “Vienna Lime” (Kremer Pigmente GmbH & Co. KG, Aichstetten, Germany), and rinsed with deionized water. Cu sheets were then activated with Pd(II) ions by immersion in 0.5 g·L^−1^ PdCl_2_ solution for 10 s and again rinsed with deionized water. Activated Cu sheets were placed into the electroless cobalt plating bath containing 0.05 M cobalt sulfate (CoSO_4_), 0.05 M morpholine borane (C_4_H_8_ONH·BH_3_), and 0.2 M glycine (NH_2_CH_2_COOH). The bath operated at pH 7 at a temperature of 30 °C for 30 min. The thickness of the pure Co coating was determined gravimetrically and it was found to be ca. 1 μm. The deposition conditions for the rest of the coatings are presented in Table 1. Salts used were Na_2_MoO_4_·2H_2_O (≥99.5%), FeSO_4_·7H_2_O (≥99%), MnSO_4_·H_2_O (≥99%), and NiSO_4_·6H_2_O (≥98%), all from Sigma–Aldrich (Taufkirchen, Germany).

### 2.2. Characterization of CoM (M = Fe, Mn, Mo), Co and Ni Coatings

The morphology and composition of the prepared coatings were investigated by scanning electron microscopy (SEM) using a SEM/FIB workstation Helios Nanolab 650 (Hillsboro, OR, USA) with an energy dispersive X-ray (EDX) spectrometer INCA Energy 350 X-Max 20 (Oxford Instruments, Abingdon, UK).

The coatings’ composition and structure were further confirmed by X-ray diffraction (XRD) analysis with diffraction data collected using Rigaku Ultima IV diffractometer in Bragg–Brentano geometry over the scattering angle 2θ range 20–80°.

Mass of the elements and metal loadings were determined by inductively coupled plasma optical emission spectrometry (ICP–OES) analysis. Prior to the analysis, all the prepared coatings were dissolved in HCl solution and diluted up to 10 mL. The ICP–OES spectra were recorded using an Optima 7000DV spectrometer (Perkin Elmer, Waltham, MA, USA) at wavelengths of λ_Co_ 228.616 nm, λ_Co_ 238.892 nm, λ_B_ 249.677 nm, λ_Mo_ 202.031 nm, λ_Mo_ 203.845 nm, λ_Fe_ 238.204 nm, λ_Mn_ 257.610 nm, and λ_Ni_ 231.604 nm.

### 2.3. Electrochemical Measurements

All electrochemical measurements were done using Ivium V01107 potentiostat (Eindhoven, The Netherlands) in a three-electrode system with saturated calomel (SCE) as a reference, graphite rod as counter and CoM (M = Fe, Mn, Mo), Co or Ni as the working electrode, in 1 M potassium hydroxide (KOH) as supporting electrolyte. All potentials in this work were converted to the reversible hydrogen electrode (RHE) scale using the following equation: E_RHE_ = E_SCE_ + 0.242 V + 0.059 V × pH_solution_. Current densities were calculated using the electrodes’ geometric area of 1 cm^2^.

CVs were recorded from 1.02 to 1.22 V, at different polarization rates in the range from 5 to 100 mV·s^−1^ in 1 M KOH saturated with nitrogen (N_2_).

HER polarization curves were recorded from the open circuit potential (OCP) to −0.38 V at a polarization rate of 5 mV·s^−1^. HER electrochemical impedance spectroscopy (EIS) analysis was conducted in the frequency range of 100 kHz to 0.1 Hz, with 5 mV amplitude at a potential of −0.33 V.

OER polarization curves were recorded from OCP to 2.12 V at a polarization rate of 5 mV·s^−1^. OER EIS measurements were done at a potential of 1.67 V with an amplitude of 5 mV in the 100 kHz–0.1 Hz range.

Polarization curves and EIS measurements were recorded at several temperatures from 25 to 85 °C, setting the temperature with a water jacket connected to a Haake water bath.

Stability was studied by recording chronoamperometry (CA) curves under HER (potential of −0.24 V) and under OER (potential of 1.67 V) conditions for 1 h for all coatings and then for 24 h for the coatings that showed the highest HER/OER activity.

## 3. Results

### 3.1. Characterization of Coatings

Mass of the elements deposited onto Cu substrate surface and their loadings, determined by ICP–OES analysis, are given in Table 2. It can be seen that the formed Co, CoMo, CoFe, and CoMn coatings contained more than 90 wt.% of Co and between 0.64–1.8 wt.% of B. In the case of Ni coating, it contained more than 97 wt.% of Ni and ~3 wt.% of boron (B).

**Figure 1:**
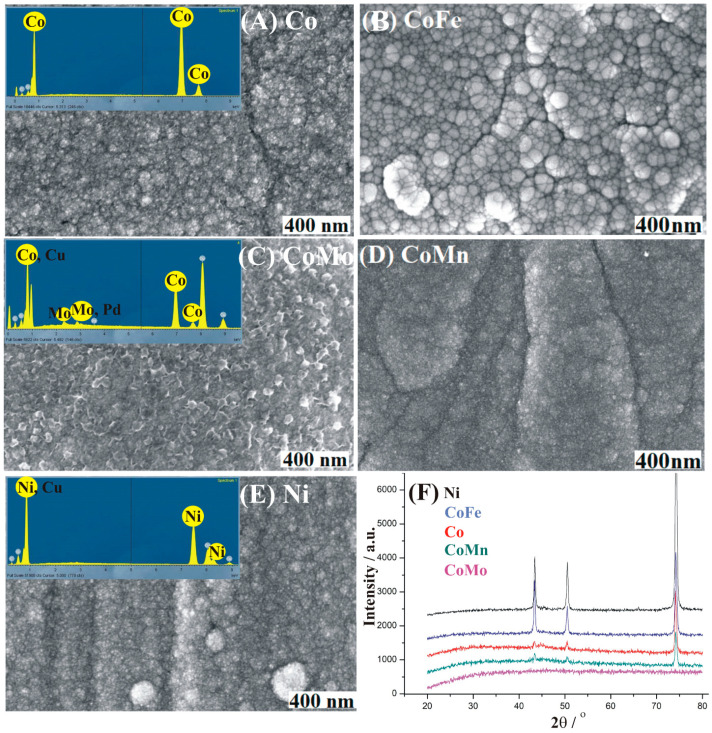
(**A**–**E**) SEM images (with EDX spectra in inset of (**A**,**C**,**E**)) of the five prepared coatings and (**F**) their XRD patterns.

SEM analysis revealed somewhat different morphology of the prepared coatings (Figure 1A–E), with EDX analysis verifying uniform distribution of components. Namely, layers of the prepared catalysts were polycrystalline, but with particles of different sizes exhibiting agglomerates of oval structure. Surface morphology plays an important role in electrocatalysis as it determines both the contact surface area as well as the number of edge active sites where the charge distribution is localized [35]. Furthermore, it plays an important role in the generation of H_2_/O_2_ gas bubbles that might block access to the active sites [36]. In the competition between the nucleation and growth of gas bubbles, a flat surface will favor the growth process leading to the formation of large bubbles, while cracks will limit the growth of O_2_ bubbles within them [36,37].

The compositions of the five coatings were further investigated by XRD analysis (Figure 1F). In case of the Ni and CoFe coatings, with low metal loading, the peaks related to the Cu substrate are also visible at 2θ of ca. 43.5, 50.5 and 74.2°, overlapping with the reflections from the Ni (111), (200) and (220) planes, respectively, and Co (002), (101) and (110) planes, respectively [5].

Electrochemical characterization of coatings was conducted by recording CVs in N_2_-saturated 1 M KOH at different polarization rates (not shown). These results were used for the investigation of charge storage capability of the studied coatings, i.e., for determination of their double-layer capacitance (C_dl_), which is proportional to their electrochemical active surface area (ECSA) [38,39,40]. Namely, adsorption of H and O (underpotential deposition) on the electrode material’s surface is a requirement for HER and OER, respectively [41]. CVs at different polarization rates indicate the rate capability of an electrode material, i.e., its efficiency to adsorb a substantial H/O amount and desorb with a high coulombic efficiency. C_dl_ was found to decrease in order CoMo (21.2 µF·cm^−2^) > Ni (21.0 µF·cm^−2^) > CoFe (6.2 µF·cm^−2^) > CoMn (3.0 µF·cm^−2^) > Co (2.1 µF·cm^−2^), with a value of 20 µF·cm^−2^ being reported as average C_dl_ value of a smooth metal surface [42]. C_dl_ values several times higher for CoMo and Ni than C_dl_ values of CoFe, CoMn, and Co coatings reflected the notably higher number of active sites at the former that could take part in the adsorption processes and lead to higher HER electrocatalytic activity. Nevertheless, the electrocatalytic reactivity of the active sites depends on their accessibility as well as their oxidation state.

### 3.2. Hydrogen Evolution Reaction Study

**Figure 2:**
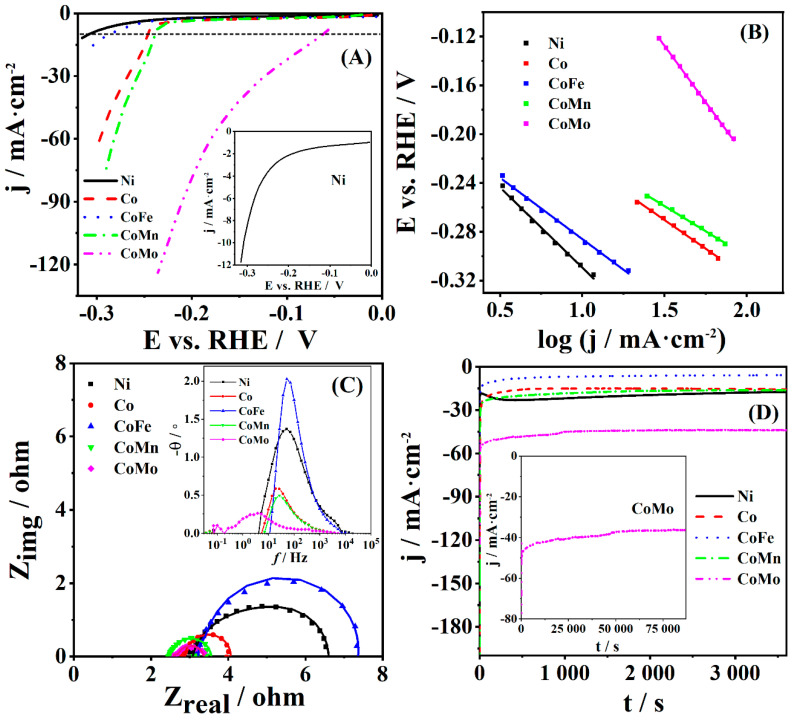
(**A**) Hydrogen evolution reaction (HER) polarization curves (iR-corrected) of five studied coatings at 5 mV·s^−1^ with (**B**) corresponding Tafel plots, (**C**) Nyquist plots at −0.33 V (amplitude of 5 mV in the 100 kHz–0.1 Hz range) with the corresponding Bode plots in the inset, and (**D**) chronoamperometric curves at −0.24 V for 1 h with the chronoamperometric curve of CoMo at the same potential for 24 h (inset). All measurements were done in 1 M KOH.

HER activity of studied coatings was evaluated in 1 M KOH by LSV method. CoMo gave the highest current density (j), followed by CoMn and Co coatings, and then by CoFe and Ni with notably lower current density during HER (Figure 2A). For instance, the current densities of −124.2, −74.1, −67.1, −16.6 and −9.5 mA·cm^−2^ were reached at −0.3 V using CoMo, CoMn, Co, CoFe, and Ni coatings, respectively. Overpotential to reach current density of 10 mA cm^−2^ (η_10_) was found to be as low as 61 mV for CoMo and increased in the order CoMo (61 mV) < CoMn (238 mV) < Co (246 mV) < CoFe (285 mV) < Ni (308 mV).

The HER kinetics was explored by Tafel analysis (Figure 2B), i.e., determination of Tafel slope (b) as a measure of a rate at which current density increases with the increase of overpotential, and exchange current density (j_0_), reflecting the electrode’s intrinsic activity for HER. Tafel slope values were found to be 83, 94, 100, 131, and 184 mV·dec^−1^ (Table 3) for CoMn, Co, CoFe, Ni, and CoMo, respectively. The exchange current density (j_0_) was calculated for HER at all five coatings by extrapolation of the Tafel plots, η vs. log j. Thus, j_0_ of 0.31, 0.04, 0.04, 0.02 and 0.01 mA·cm^−2^ were calculated for CoMo, Ni, Co, CoMn, and CoFe, respectively (Table 3). It is worth noting that the j_0_ value determined for HER at CoMo coating was ca. seven times higher than that determined for the rest of the studied coatings.

**Scheme 1:**
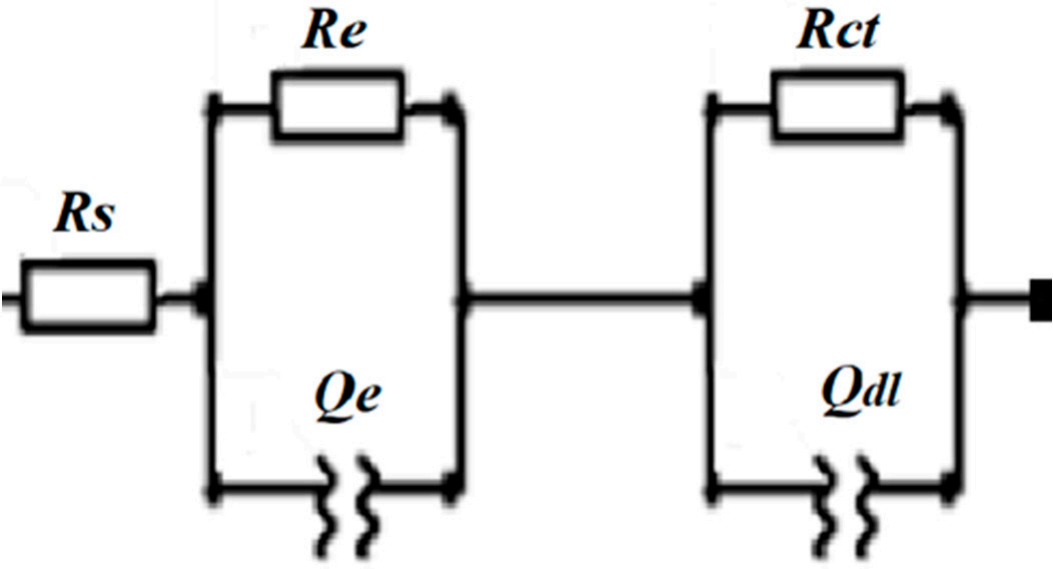
Equivalent electric circuit used to fit the electrochemical impedance spectroscopy (EIS) data, where *R_s_* is the electrolyte resistance, *R_e_* is the electronic resistance of the material and the associated capacitance *R_ct_* is the resistance of the charge transfer reaction, *Q_e_* is the constant phase element, and *Q_dl_* is an ideal double-layer capacitor.

The EIS measurements were used for additional investigation of the coating/electrolyte interface and the related processes occurring at the coating surface under HER conditions in 1 M KOH. Figure 2C shows the Nyquist and Bode (inset) plots of the five coatings. The solution resistance (*R_s_*) (2.48–3.20 Ω range), as well as the charge transfer resistance (*R_ct_*) (1.08–2.80 Ω range), were calculated using the equivalent circuit presented in Scheme 1 and found to be comparable for the studied coatings (Table 4).

Another crucial criterion for an advanced electrode material is its electrochemical stability. Chronoamperometric measurements with five coatings were done in 1 M KOH at −0.24 V for 1 h. CA results confirmed the result of LSV analysis in terms of CoMo giving the highest current density (−50.2 mA·cm^−2^ at 200 s) during HER (Figure 2D). More than two times lower current density was recorded with Ni (−22.4 mA·cm^−2^), CoMn (−21.7 mA·cm^−2^) and Co (18.2 mA·cm^−2^) coatings and notably lower value with CoFe (−5.7 mA·cm^−2^). Activity for HER of all coatings (in terms of the recorded current density) was found to be stable during one-hour measurement. Thus, current density under HER conditions at CoMo decreased ca. 13% after 1 h. Furthermore, CoMo showed relatively stable current density during 24 h with a decrease of ca. 18.6%. Thus, HER current density of CoMo was found to be −54.3 mA·cm^−2^ at 800 s and −44.2 mA·cm^−2^ at 86,400 s.

**Figure 3:**
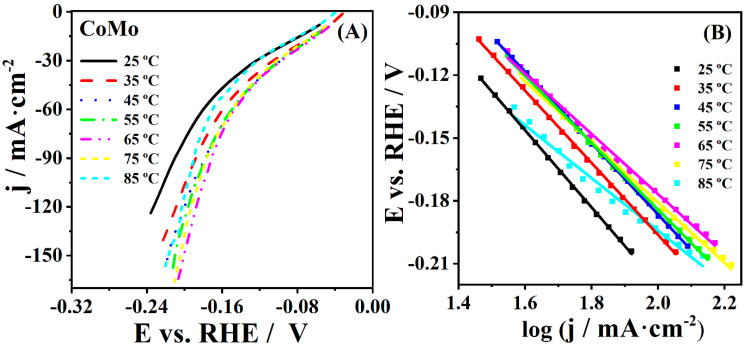
(**A**) HER polarization curves of CoMo coatings in 1 M KOH recorded at polarization rate of 5 mV·s^−1^ at different temperatures with (**B**) corresponding Tafel plots.

HER at five coatings was studied at temperatures ranging from 25 up to 85 °C. The current densities of CoMo increased from −124.2 to −164.8 mA·cm^−2^ with increasing temperature from 25 to 65 °C, respectively, and then somewhat decreased at 75 and 85 °C (e.g., to 156.3 mA·cm^−2^ at 85 °C). Ni and CoFe showed the same trend of increasing current densities with the increase of temperature, whereas CoMn and Co did not show a significant difference in current densities with increasing temperature during HER. Furthermore, Tafel slopes were calculated for HER at five studied coatings at different temperatures (Figure 3). A decrease of Tafel slope value with increasing temperature was observed, e.g., Tafel slope for HER at CoMo was determined to be 184 and 126 mV·dec^−1^ at 25 and 85 °C, respectively.

### 3.3. Oxygen Evolution Reaction

**Figure 4:**
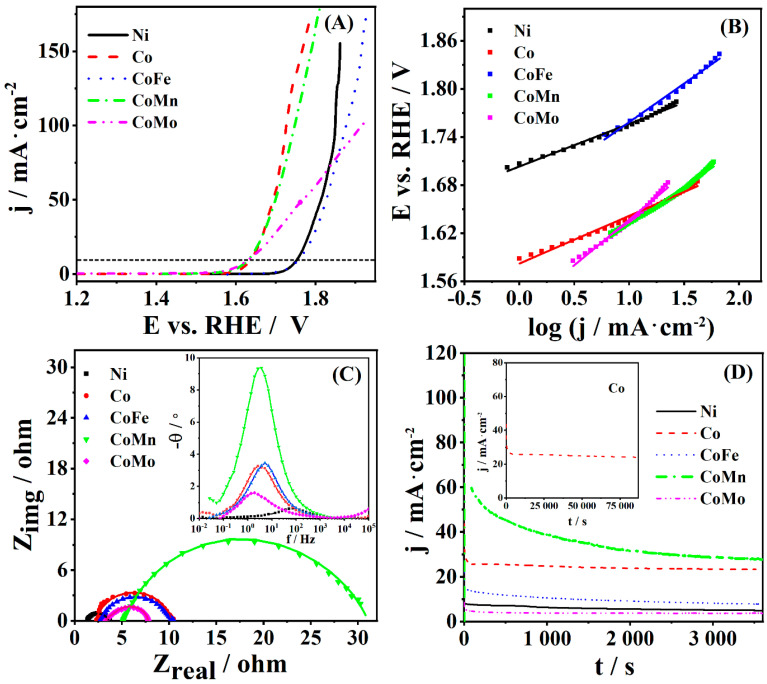
(**A**) Oxygen evolution reaction (OER) polarization curves (iR-corrected) of five studied coatings at 5 mV·s^−1^ with (**B**) corresponding Tafel plots, (**C**) Nyquist plots at 1.67 V (amplitude of 5 mV in the 100 kHz–0.1 Hz range) with the corresponding Bode plots in the inset, and (**D**) chronoamperometric curves at 1.67 V for 1 h with the chronoamperometric curve of Co coating at the same potential for 24 h (inset). All measurements were done in 1 M KOH.

The activity of CoM (M = Fe, Mn, Mo), Co, and Ni coatings for oxygen evolution reaction was also thoroughly examined in alkaline media (1 M KOH). The polarization curves of studied coatings are presented in Figure 4, where it can be observed that the highest current density during OER was recorded at Co and CoMn, followed by CoFe and Ni, and then CoMo coating. An overpotential to reach a current density of 10 mA·cm^−2^ was found to be similar for Co (438 mV), CoMn (431 mV) and CoMo (431 mV) and ca. 120 mV higher for CoFe (553 mV) and Ni (559 mV) coatings (Table 5). Furthermore, the current density of 150 mA·cm^−2^ was reached at potential increasing in the following order Co (1.77 V) > CoMn (1.79 V) > Ni (1.86 V) > CoFe (1.92 V). Current density at CoMo coating did not reach a value of 150 mA cm^−2^ in the investigated potential range; it reached a value of only 99 mA cm^−2^ at 1.90 V.

OER polarization curves were then further used for constructing the Tafel plots and calculating the Tafel slope. Tafel slope values of 53 and 60 mV·dec^−1^ were found for OER at Ni and Co, respectively. Higher values of 94, 96, and 114 mV·dec^−1^ were determined for OER at CoMn, CoFe, and CoMo, respectively.

To get a deeper insight into the OER kinetics at studied coatings, EIS analysis was also performed in 1 M KOH under the OER conditions. Figure 4C shows the Nyquist plots at 1.67 V (amplitude of 5 mV in the 100 kHz–0.1 Hz range) and the Bode plots (inset of the figure) of the five coatings with *R_ct_* determined (using the equivalent circuit presented in Scheme 1) to be 1.01, 2.24, 7.44, 7.90, and 20.16 Ω for CoMo, Ni, CoFe, Co, and CoMn coatings, respectively (Table 6). Furthermore, Co coating showed a favorable C_dl_ value of 9.6 mF.

As mentioned, the electrode’s electrochemical stability is another key criterion for the evaluation of its potential application. Figure 4D presents chronoamperometric curves of studied coatings at the potential of 1.67 V where the highest current density at 3600 s was recorded at CoMn (27.9 mA·cm^−2^) and Co (23.5 mA·cm^−2^), followed by CoFe (7.76 mA·cm^−2^), Ni (5.08 mA·cm^−2^) and CoMo (3.48 mA·cm^−2^) coatings. Chronoamperometric curves of CoMn and CoFe coatings revealed somewhat lower stability of these two coatings as they showed a current density decrease of about 50 and 40%, respectively, when comparing the values in 180 and 3600 s. Co showed good stability during 24 h where current density decreased from 28.6 mA·cm^−2^ at 800 s to 25.5 mA·cm^−2^ at 27,700 s.

**Figure 5:**
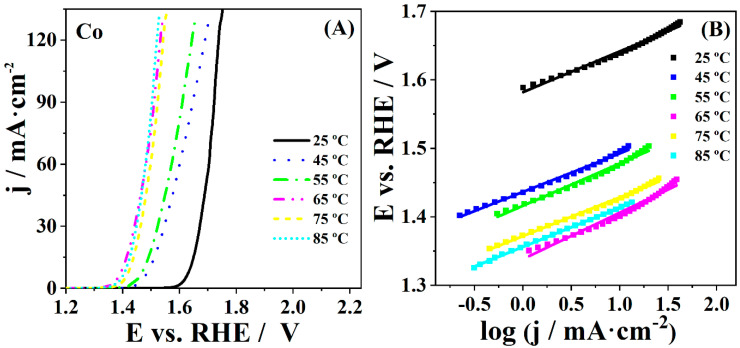
(**A**) OER polarization curves of Co coating in 1 M KOH recorded at polarization rate of 5 mV·s^−1^ at different temperatures with (**B**) corresponding Tafel plots.

Additionally, the impact of temperature on OER activity of five studied coatings in 1 M KOH was thoroughly tested at temperatures ranging from 25 to 85 °C. A pronounced increase of current density with temperature was observed. Thus, the potential to reach a current density of 120 mA·cm^−2^ at Co coating decreased from 1.74 V at 25 °C to 1.52 V at 85 °C (Figure 5A). Figure 5B shows the corresponding Tafel plots of Co coating at different temperatures where Tafel slope values changed slightly; the same behavior was observed for the other coatings as well.

## 4. Discussion

HER in alkaline solution proceeds by the following three steps:

M + H_2_O + e^−^ ↔ MH_ads_ + OH^−^ (Volmer step, b = 120 mV·dec^−1^), (1)

MH_ads_ + H_2_O + e^−^ ↔ H_2_ + M + OH^−^ (Heyrovsky step, b = 40 mV·dec^−1^), (2)

MH_ads_ + MH_ads_ ↔ H_2_ + 2M (Tafel step, b = 30 mV·dec^−1^). (3)

The Volmer step represents electrosorption of the hydrogen proton (H^+^) to form the surface adsorbed hydrogen atom (MH_ads_) on the active metal sites (M), the Heyrovsky step is the electrochemical desorption of MH_ads_ to form H_2_, and the Tafel step represents recombination of two MH_ads_ on the metal surface to form H_2_ [32,33]. Tafel slope values suggest that HER at CoMn, Co, and CoFe proceeds by the Volmer–Heyrovsky mechanism where the electrochemical desorption of the MH_ads_ is the rate-limiting step [32,34], while during HER at Ni and CoMo, the Volmer step, i.e., electrochemical sorption of H^+^, represents the rate-determining step. It could be seen that the determined Tafel slope values deviated from the theoretical ones toward higher values that might have been caused by the formation of surface oxides of lower conductivity compared to the metallic surface [34].

The notably higher double-layer capacitance of CoMo coating could account for its higher activity toward HER compared to the other coatings. Furthermore, the overpotential value to reach the current density of 10 mA·cm^−2^ determined for CoMo was lower than the values of 73 and 288 mV reported for S-CoO nanorods [19,20] and Co_3_O_4_–CuO electrocatalyst [19,20], respectively (Table 7). It was also lower than the overpotential of 163.0 mV to achieve a current density of 10 mA·cm^−2^ for HER at CoP–FeP in alkaline electrolyte solution [2]. Furthermore, it was lower than the overpotential of 119 and 297 mV at 10 mA·cm^−2^ at Mo_2_C@C and Ni foam, respectively [37,38].

Obtained results showed that the combination of Co as right-hand side transition metal with Mo as left-hand side transition metal results in enhancement of the coating’s intrinsic activity. Material electrocatalytic activity for HER is governed by the H adsorption free energy [41]. The well-known volcano plot indicates noble metals as the best electrocatalysts for HER, while the activity of transition metals can be improved by combining multiple metals with different, weak and strong, bonds with hydrogen [43]. Consequently, metal forming a strong bond with H initiates the H adsorption, with the possibility of H_ads_ moving via surface diffusion. Metal forming a weak bond with H then facilitates the H_2_ generation and its release from the electrode surface. Furthermore, the presence of a second metal of different size changes the lattice structure, which can result in the generation of active sites. Finally, a combination of two transition metals can improve the electrochemical stability compared to the components [41].

As for the state of the surface, it has been suggested that the presence of oxides on the metal surface is crucial for HER activity due to the oxides’ affinity to form OH_ads_ and thus promote water splitting [35]. Formed OH species adsorb on the metal oxide, while H atoms formed adsorb at the neighboring metal active sites. Thus, adequate tailoring metal/metal oxide interfaces can lead to a bifunctional HER mechanism.

An OER Tafel slope value lower than 60 mV·dec^−1^ suggests a four-electron transfer determining step while a value higher than 60 mV·dec^−1^ suggests a three-electron transfer determining step [36], with increased multiple electron transfer process (case of Ni and Co), indicating better electrocatalytic performance of a coating. Tafel slope for OER at NiFeMo oxide catalyst was reported to be 64 mV·dec^−1^ [38] and at NiCo_2_O_4_ 89 mV·dec^−1^ [40] in 0.1 M KOH, i.e., higher than the values for herein tested Co coating (Table 8).

Overpotential at a current density of 10 mA·cm^−2^ values are somewhat higher than those given in the literature reports for OER at different transition metal-based electrodes (Table 8). Thus, CoMn, CoMo, and Co can be seen as good materials for OER, and CoFe and Ni as satisfactory ones [42]. Transition metals’ activity towards OER is governed, among other factors, by the number and accessibility of active sites, their oxidation state (+2, +3, or +4), 3D electrons number, and surface oxygen binding energy [48]. Namely, OER is reported ideally not to be a surface reaction, but to occur in a ca. 10 nm layer of the electrode material [37]. Furthermore, it has been reported that parallel with the OER occurs the phase conversion of Co-based materials (metal to oxides, oxides to hydroxides or oxyhydroxides), taking an active role during OER. Active sites in their high oxidation state, with the transition occurring at potentials lower than that of OER via a pseudocapacitive behavior, will favor activity for the OER [37]. Incorporation of a second transition metal typically should boost the OER activity by altering intermediate bonds and electronic structure, and, thus, increasing the electric conductivity [49]. The strength of the OH_ads_–M^2+δ^ bond increases in the order Ni < Co < Fe < Mn [50].

## 5. Conclusions

CoM (M = Fe, Mo, Mn), Co, and Ni coatings were prepared by electroless deposition with SEM revealing somewhat different morphology. The effect of combining Co with left-hand side transition metals (Fe, Mo, Mn) on the electrocatalytic activity for water electrolysis, i.e., for both HER and OER in alkaline media, was evaluated. CoMo coating gave the highest current densities as well as exchange current density during HER in 1 M KOH, showing a faster charge transfer through the metal–solution interface during HER. Low charge transfer resistance in the case of CoMo was confirmed by EIS analysis. Furthermore, CoMo showed the highest value of double-layer capacitance, reflecting the highest number of active sites.

On the other hand, Co and CoMn coating showed the highest current density during OER with ca. 120 mV lower overpotential to reach a current density of 10 mA·cm^−2^, compared to CoFe and Ni coatings. Co coating showed faster HER kinetics in terms of low Tafel slope (60 mV·dec^−1^) resulting from lower charge transfer resistance and higher double-layer capacitance.

Stability tests under HER as well as OER conditions in alkaline media revealed high stability of coatings studied.

## Figures and Tables

**Table 1:** Deposition conditions during the preparation of the herein studied coatings.

	Deposition Conditions										
Coating	CoSO_4_/M	Morpholine Borane/M	Glycine/M	Mo^6+^/mM	Fe^2+^/mM	Mn^2+^/mM	Ni^2+^/mM	Trisodium Citrate/mM	pH	T/°C	t/min
Co	0.05	0.05	0.2	-	-	-	-	-	7	30	30
CoMo	0.1	0.2	-	1	-	-	-	-	7	60	30
CoFe	0.07	0.06	0.2	-	5	-	-	18	7	50	30
CoMn	0.07	0.06	0.2	-	-	5	-	18	7	50	30
Ni	-	0.02	0.2	-	-	-	50	40	7	30	30

**Table 2:** Mass of the elements and total metal loading in the coatings as determined by inductively coupled plasma optical emission spectrometry (ICP–OES) analysis.

Coating	Mass of Element/mg						Total Metal Loading/mg·cm^−2^
	Co	B	Mo	Fe	Mn	Ni	
Co	2.027	0.01413	-	-	-	-	1.02057
CoMo	3.198	0.06367	0.2275	-	-	-	1.74459
CoFe	0.5472	0.00384	-	0.05282	-	-	0.30193
CoMn	3.064	0.02331	-	-	0.0017	-	1.54451
Ni	-	0.01591	-	-	-	0.525	0.27046

**Table 3:** Electrochemical performance of the tested coatings toward HER in alkaline media.

Coating	Tafel Slope (mV·dec^−1^)	j_0_ (mA·cm^−2^)	η_10_ (mV)	*R_ct_* (Ω)	Electrolyte
CoFe	100	0.02	285	2.10	1 M KOH
CoMn	83	0.02	238	1.03	1 M KOH
CoMo	184	0.31	61	1.15	1 M KOH
Co	94	0.04	246	2.80	1 M KOH
Ni	131	0.04	308	1.07	1 M KOH

**Table 4:** EIS parameters of the herein prepared coatings in 1 M KOH at –0.33 V (amplitude of 5 mV in the 100 kHz–0.1 Hz range).

Coating	*R_s_* (Ω)	*R_e_* (Ω)	*R_ct_* (Ω)	*Q_e_* (mF)	*Q_dl_* (mF)
CoMo	2.56	6.60	1.15	100.0	40.0
CoFe	3.20	3.90	2.10	0.4	0.4
CoMn	2.48	0.02	1.03	637.4	6.8
Co	2.85	0.43	2.80	16.9	6.9
Ni	3.06	2.42	1.07	1.8	3.3

*R_s_*—the electrolyte resistance, *R_e_*—the electronic resistance of the material and the associated capacitance, *R_ct_*—resistance of the charge transfer reaction, *Q_e_*—the constant phase element, *Q_d_*—the ideal double-layer capacitor.

**Table 5:** Electrochemical performance of the tested coatings toward OER in alkaline media.

Coating	Tafel Slope (mV dec^−1^)	η_10_ (mV)	*R_ct_* (Ω)	Electrolyte
CoFe	96	559	7.44	1 M KOH
CoMn	94	431	20.16	1 M KOH
CoMo	114	431	1.01	1 M KOH
Co	60	438	7.9	1 M KOH
Ni	53	553	2.24	1 M KOH

**Table 6:** EIS parameters of OER at the studied coatings in 1 M KOH at 1.67 V (amplitude of 5 mV in the 100 kHz–0.1 Hz range).

Coating	*R_s_* (Ω)	*R_e_* (Ω)	*R_ct_* (Ω)	*Q_e_* (mF)	*Q_dl_* (mF)
CoMn	5.16	25.5	20.2	3.0	1.0 × 10^−10^
CoFe	2.38	3.10	7.44	1.0 × 10^−10^	7.1
CoMo	3.29	3.44	1.01	32	25
Co	0.45	1.82	7.90	8.3 × 10^−3^	9.6
Ni	0.02	1.29	2.24	1.7 × 10^−4^	4.3

*R_s_*—the electrolyte resistance, *R_e_*—the electronic resistance of the material and the associated capacitance, *R_ct_*—resistances of the charge transfer reaction, *Q_e_*—the constant phase element, *Q_dl_*—the ideal double-layer capacitor, *W*—Warburg element.

**Table 7:** Electrochemical performance of herein tested coatings towards HER in alkaline media and comparison with that of transition metal-based electrodes reported in the literature.

Electrode	Tafel Slope (mV·dec^−1^)	j_0_ (mA·cm^−2^)	η_10_ (mV)	*R_ct_* (Ω)	Electrolyte	Ref.
CoFe	100	0.02	285	2.10	1 M KOH	This work
CoMn	83	0.02	238	1.03	1 M KOH	This work
CoMo	184	0.31	61	1.15	1 M KOH	This work
Co	94	0.04	246	2.80	1 M KOH	This work
Ni	131	0.04	308	1.07	1 M KOH	This work
Co_3_O_4_-CuO	65	-	288	173.20	1 M KOH	[26]
Co_3_O_4_	94	-	-	384.50	1 M KOH	[26]
CuO	243	-	-	656.90	1 M KOH	[26]
P-CoO NRs *	164	-	208	-	1 M KOH	[25]
CoP-FeP	51.2	-	163	141	1 M KOH	[2]
Mo_2_C@C	51	-	119	-	1 M KOH	[44]
Ni-rGO/Ni foam **	108	0.5	187	32.30	1 M NaOH	[45]
Fe_1.89_Mo_4.11_O_7_/MoO_2_	79	-	197	-	1 M KOH	[46]
Co_9_S_8_-MoS_2_@3DC ***	83.6	-	177	41.42	1 M KOH	[47]
MoS_2_@3DC	102.8	-	252	316.3	1 M KOH	[47]
Co_9_S_8_@3DC	97.1	-	242	162.4	1 M KOH	[47]

* NRs—nanorods, ** rGO—the reduced graphene oxide, *** 3DC—the three-dimensional interconnected hierarchical pore carbon.

**Table 8:** Electrochemical performance of herein tested coatings toward OER in alkaline media and comparison with that of transition metal-based electrodes reported in the literature.

Electrode	Tafel Slope (mV·dec^−1^)	η_10_ (mV)	*R_ct_* (Ω)	Electrolyte	Ref.
CoFe	96	559	7.44	1 M KOH	This work
CoMn	94	431	20.16	1 M KOH	This work
CoMo	114	431	1.01	1 M KOH	This work
Co	60	438	7.9	1 M KOH	This work
Ni	53	553	2.24	1 M KOH	This work
Co_3_Fe-MOF	55.6	314	-	1 M KOH	[51]
NiCoFeO_x_ NSs/NF basal growth NHS	61	260	1.52	1 M KOH	[3]
Ultrathin NiCoFeOx NSs/NF	84	305	-	1 M KOH	[3]
P-NMO	70.3	370 at 5 mA·cm^−2^	-	1 M KOH	[31]
NMO	80.6	440 at 5 mA·cm^−2^	-	1 M KOH	[31]
Co–P/NF-10	51.1	306	20	1 M KOH	[5]
c-NiFeMo	64	440	85	0.1 M KOH	[52]
GFC20_800_	180	-	110.3	1 M KOH	[53]
GFC20_700_	154	-	90.3	1 M KOH	[53]
NiCo_2_O_4_	89	-	-	0.1 M KOH	[54]
Co_3_O_4_	113	-	-	0.1 M KOH	[54]
Pt/C	133	-	-	0.1 M KOH	[54]

NSs—nanosheets; NF—nickel foam; NHS—nanoheterostructure; P-NMO—phosphorized NiMoO_4_; NMO—NiMoO_4_; GFC20_800_ and GFC20_700_—gadolinium ferrite perovskite doped with divalent cation Cu^2+^, GdFe_1-x_Cu_x_O_3_ (0 ≤ x ≤ 0.3), calcination temperature of 800 and 700 °C, respectively.

## Data Availability

The data presented in this study are available on request from the corresponding author.
